# Supplementary material for: Multiple Myeloma Derived Extracellular Vesicle Uptake by Monocyte Cells Stimulates IL-6 and MMP-9 Secretion and Promotes Cancer Cell Migration and Proliferation
Source: Cancers (Basel). 2024 Feb 29;16(5):1011. doi: 10.3390/cancers16051011 (PMC10930391; doi:10.3390/cancers16051011)
Supplement: Supplementary file 1 [file cancers-16-01011-s001.zip › cancers-2862466-supplementary.pptx]

## Slide 1
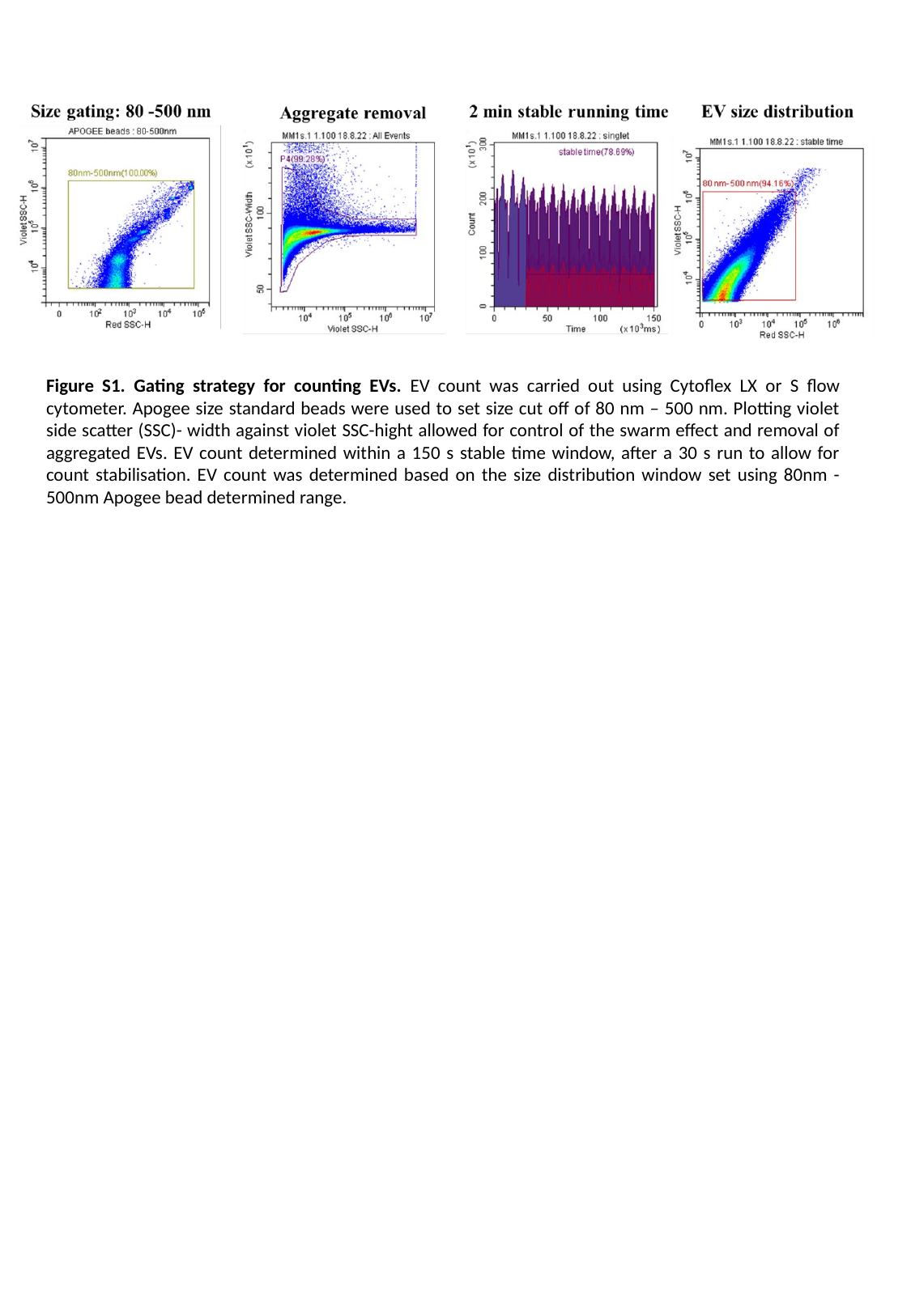

Figure S1. Gating strategy for counting EVs. EV count was carried out using Cytoflex LX or S flow cytometer. Apogee size standard beads were used to set size cut off of 80 nm – 500 nm. Plotting violet side scatter (SSC)- width against violet SSC-hight allowed for control of the swarm effect and removal of aggregated EVs. EV count determined within a 150 s stable time window, after a 30 s run to allow for count stabilisation. EV count was determined based on the size distribution window set using 80nm -500nm Apogee bead determined range.

## Slide 2
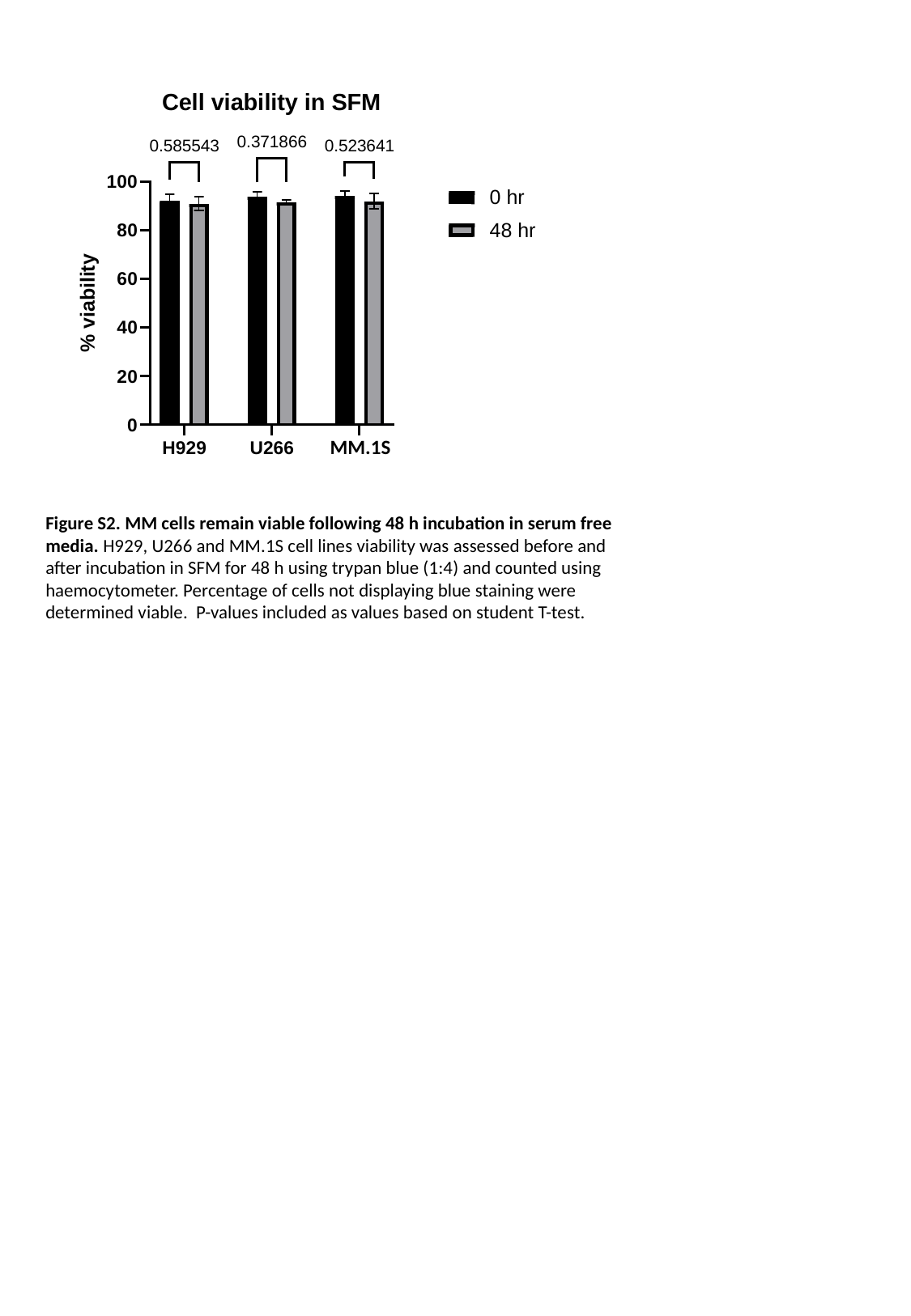

MM.1S
Figure S2. MM cells remain viable following 48 h incubation in serum free media. H929, U266 and MM.1S cell lines viability was assessed before and after incubation in SFM for 48 h using trypan blue (1:4) and counted using haemocytometer. Percentage of cells not displaying blue staining were determined viable. P-values included as values based on student T-test.

## Slide 3
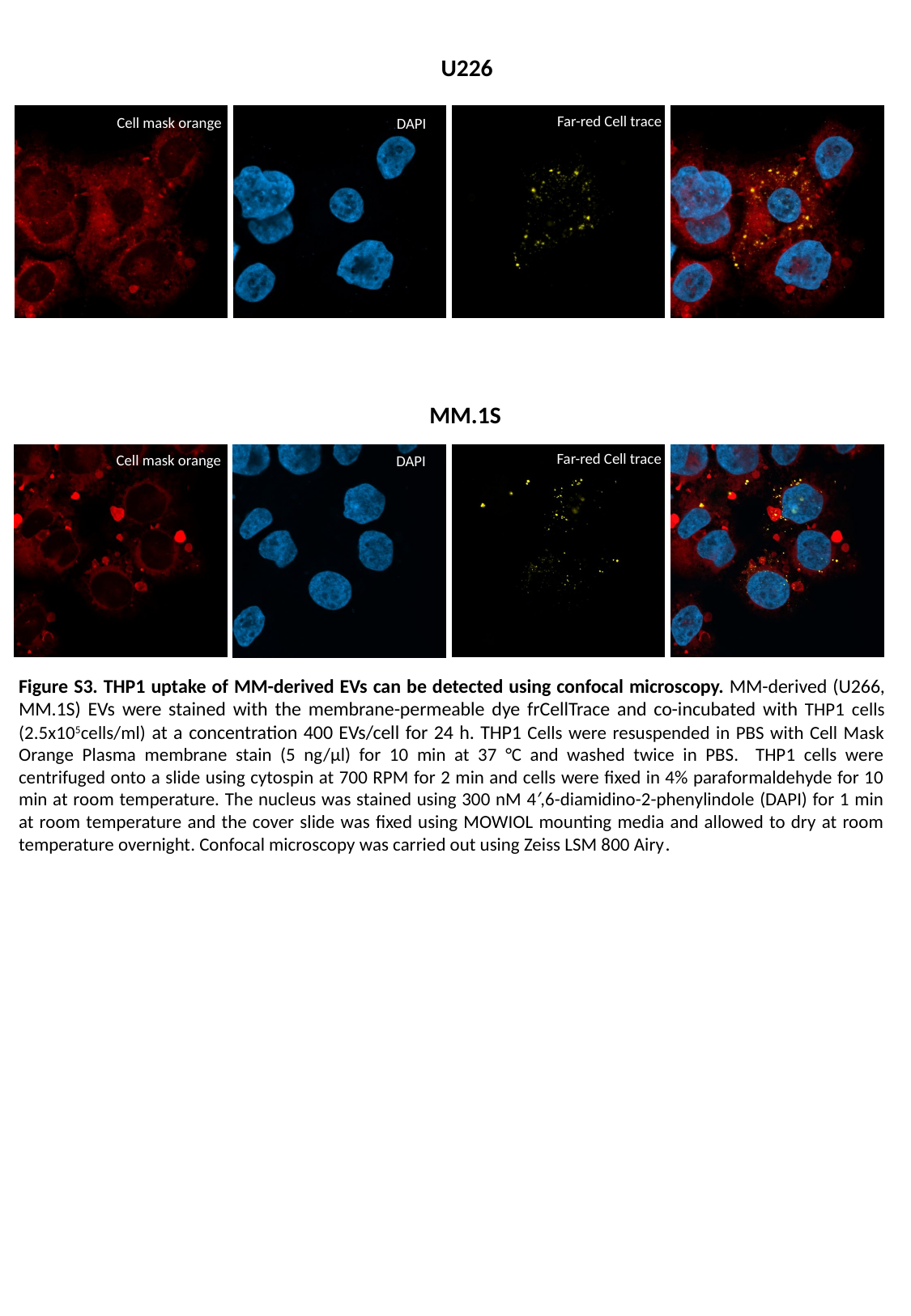

U226
Far-red Cell trace
Cell mask orange
DAPI
MM.1S
Far-red Cell trace
Cell mask orange
DAPI
Figure S3. THP1 uptake of MM-derived EVs can be detected using confocal microscopy. MM-derived (U266, MM.1S) EVs were stained with the membrane-permeable dye frCellTrace and co-incubated with THP1 cells (2.5x10^5^cells/ml) at a concentration 400 EVs/cell for 24 h. THP1 Cells were resuspended in PBS with Cell Mask Orange Plasma membrane stain (5 ng/µl) for 10 min at 37 °C and washed twice in PBS. THP1 cells were centrifuged onto a slide using cytospin at 700 RPM for 2 min and cells were fixed in 4% paraformaldehyde for 10 min at room temperature. The nucleus was stained using 300 nM 4′,6-diamidino-2-phenylindole (DAPI) for 1 min at room temperature and the cover slide was fixed using MOWIOL mounting media and allowed to dry at room temperature overnight. Confocal microscopy was carried out using Zeiss LSM 800 Airy.

## Slide 4
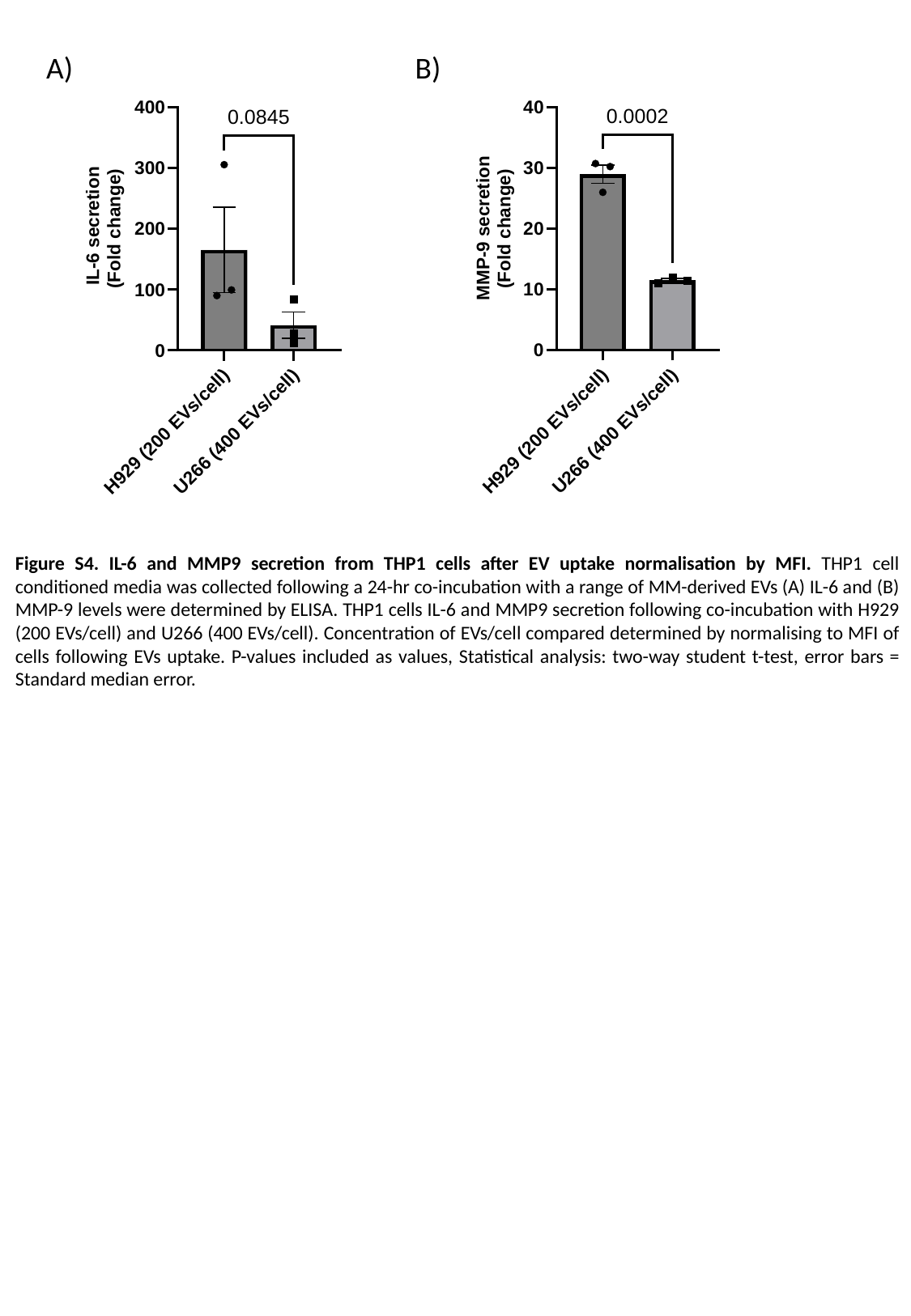

A)
B)
Figure S4. IL-6 and MMP9 secretion from THP1 cells after EV uptake normalisation by MFI. THP1 cell conditioned media was collected following a 24-hr co-incubation with a range of MM-derived EVs (A) IL-6 and (B) MMP-9 levels were determined by ELISA. THP1 cells IL-6 and MMP9 secretion following co-incubation with H929 (200 EVs/cell) and U266 (400 EVs/cell). Concentration of EVs/cell compared determined by normalising to MFI of cells following EVs uptake. P-values included as values, Statistical analysis: two-way student t-test, error bars = Standard median error.

## Slide 5
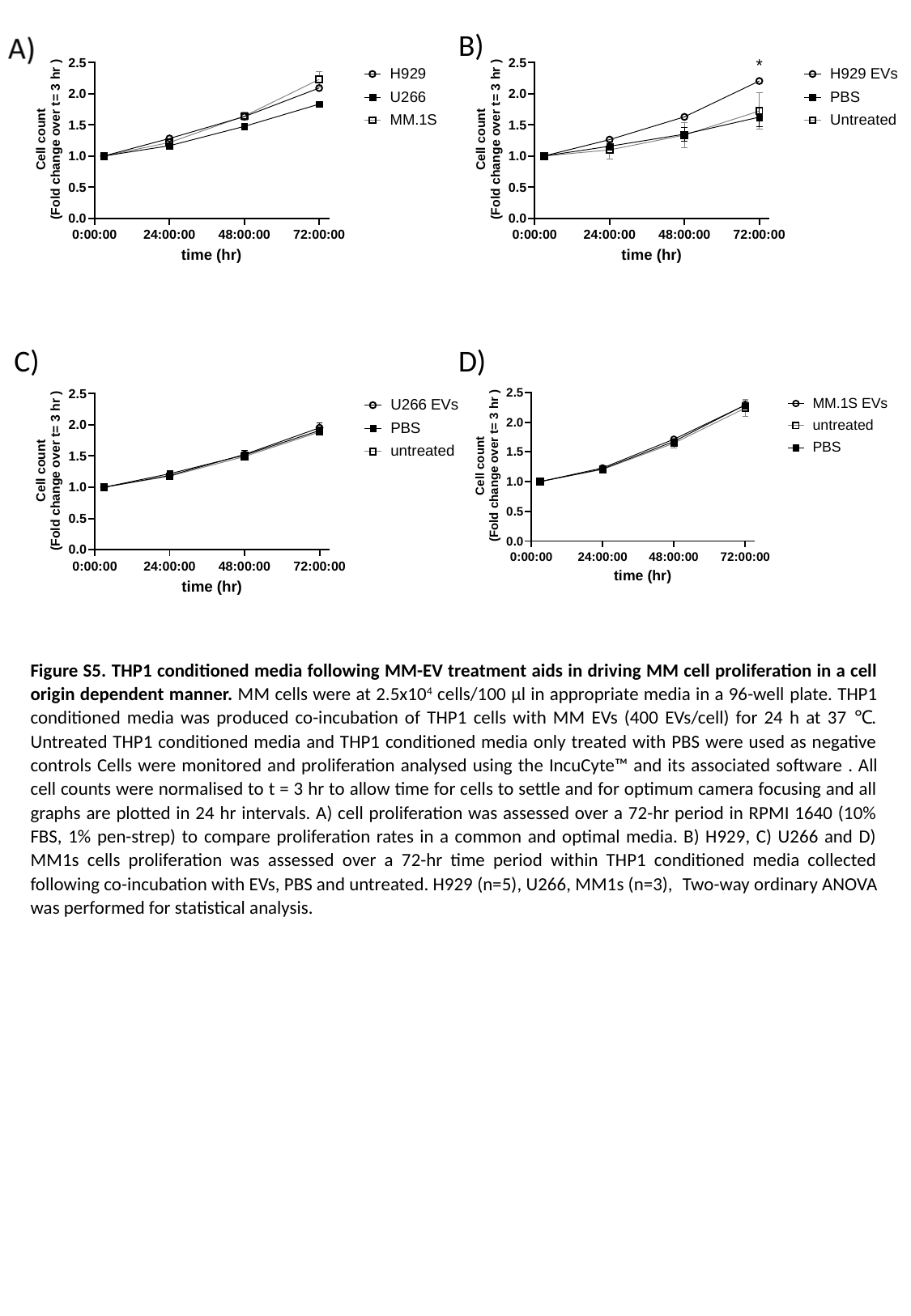

B)
C)
D)
Figure S5. THP1 conditioned media following MM-EV treatment aids in driving MM cell proliferation in a cell origin dependent manner. MM cells were at 2.5x10^4^ cells/100 µl in appropriate media in a 96-well plate. THP1 conditioned media was produced co-incubation of THP1 cells with MM EVs (400 EVs/cell) for 24 h at 37 ℃. Untreated THP1 conditioned media and THP1 conditioned media only treated with PBS were used as negative controls Cells were monitored and proliferation analysed using the IncuCyte™ and its associated software . All cell counts were normalised to t = 3 hr to allow time for cells to settle and for optimum camera focusing and all graphs are plotted in 24 hr intervals. A) cell proliferation was assessed over a 72-hr period in RPMI 1640 (10% FBS, 1% pen-strep) to compare proliferation rates in a common and optimal media. B) H929, C) U266 and D) MM1s cells proliferation was assessed over a 72-hr time period within THP1 conditioned media collected following co-incubation with EVs, PBS and untreated. H929 (n=5), U266, MM1s (n=3),  Two-way ordinary ANOVA was performed for statistical analysis.

## Slide 6
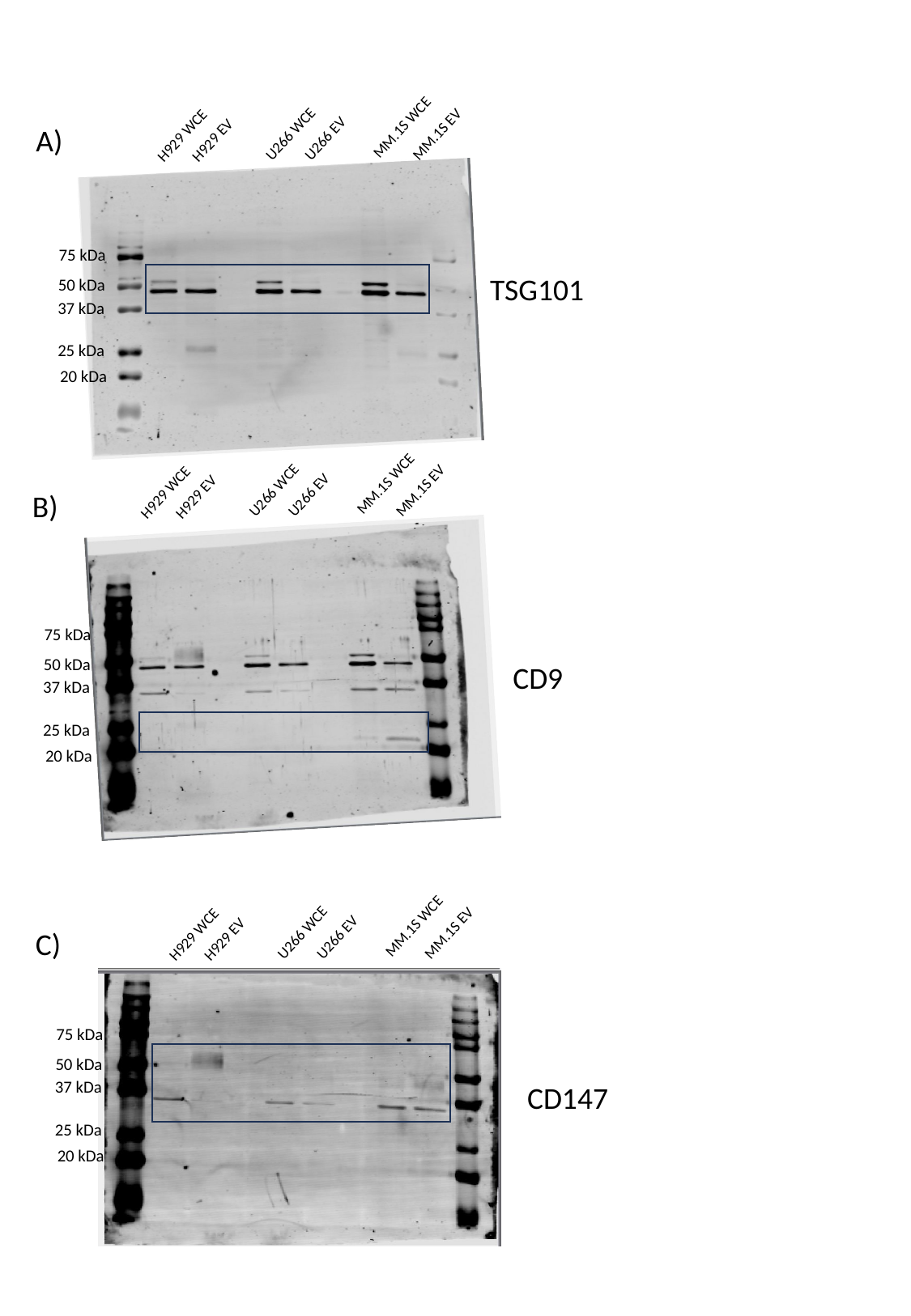

MM.1S WCE
MM.1S EV
U266 WCE
U266 EV
H929 WCE
H929 EV
A)
75 kDa
TSG101
50 kDa
37 kDa
25 kDa
20 kDa
MM.1S WCE
MM.1S EV
U266 WCE
U266 EV
H929 WCE
H929 EV
B)
75 kDa
50 kDa
CD9
37 kDa
25 kDa
20 kDa
MM.1S WCE
MM.1S EV
U266 WCE
U266 EV
H929 WCE
H929 EV
C)
75 kDa
50 kDa
37 kDa
CD147
25 kDa
20 kDa

## Slide 7
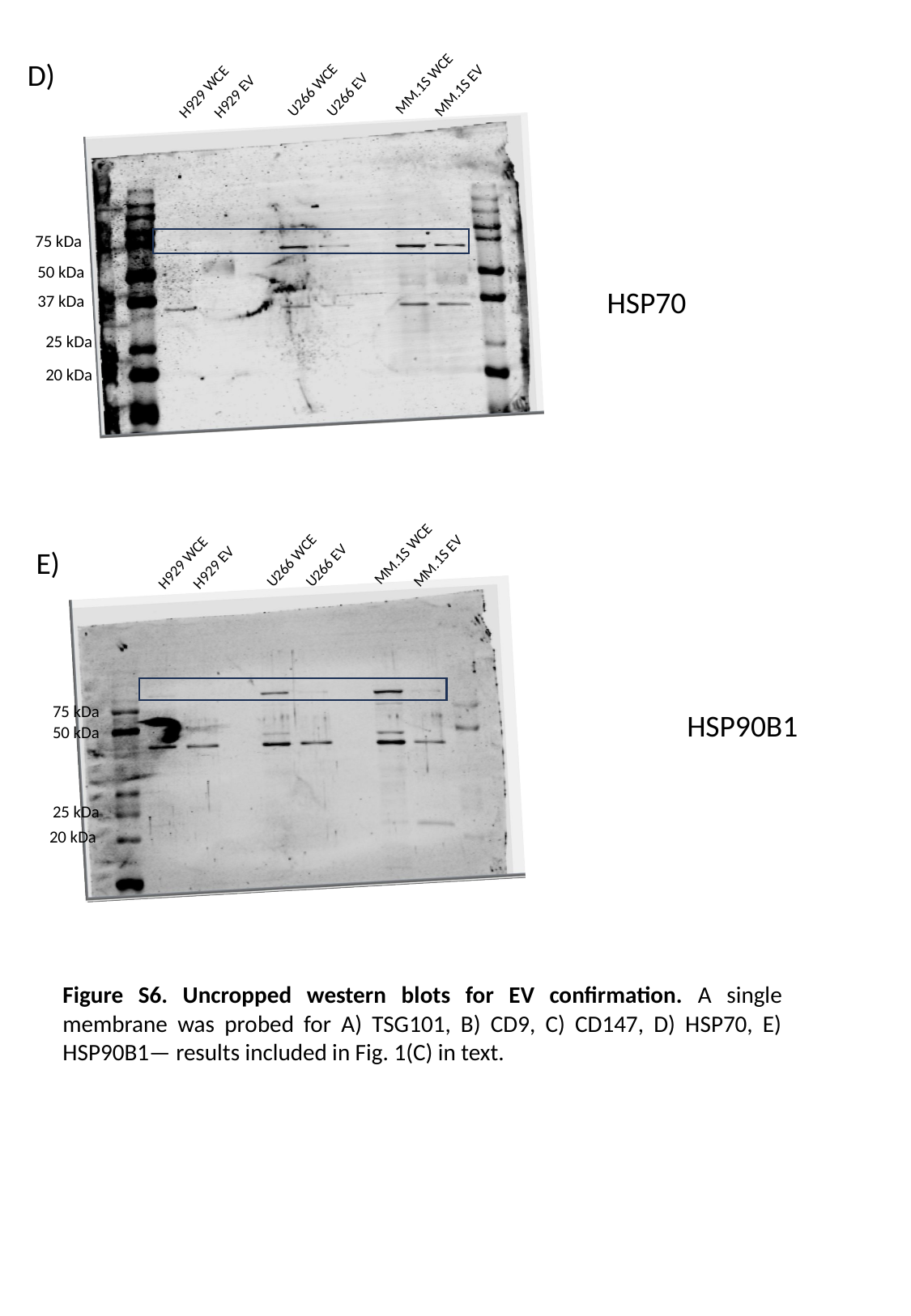

MM.1S WCE
MM.1S EV
U266 WCE
U266 EV
H929 WCE
H929 EV
D)
75 kDa
50 kDa
HSP70
37 kDa
25 kDa
20 kDa
MM.1S WCE
MM.1S EV
U266 WCE
U266 EV
H929 WCE
H929 EV
E)
75 kDa
HSP90B1
50 kDa
25 kDa
20 kDa
Figure S6. Uncropped western blots for EV confirmation. A single membrane was probed for A) TSG101, B) CD9, C) CD147, D) HSP70, E) HSP90B1— results included in Fig. 1(C) in text.

## Slide 8
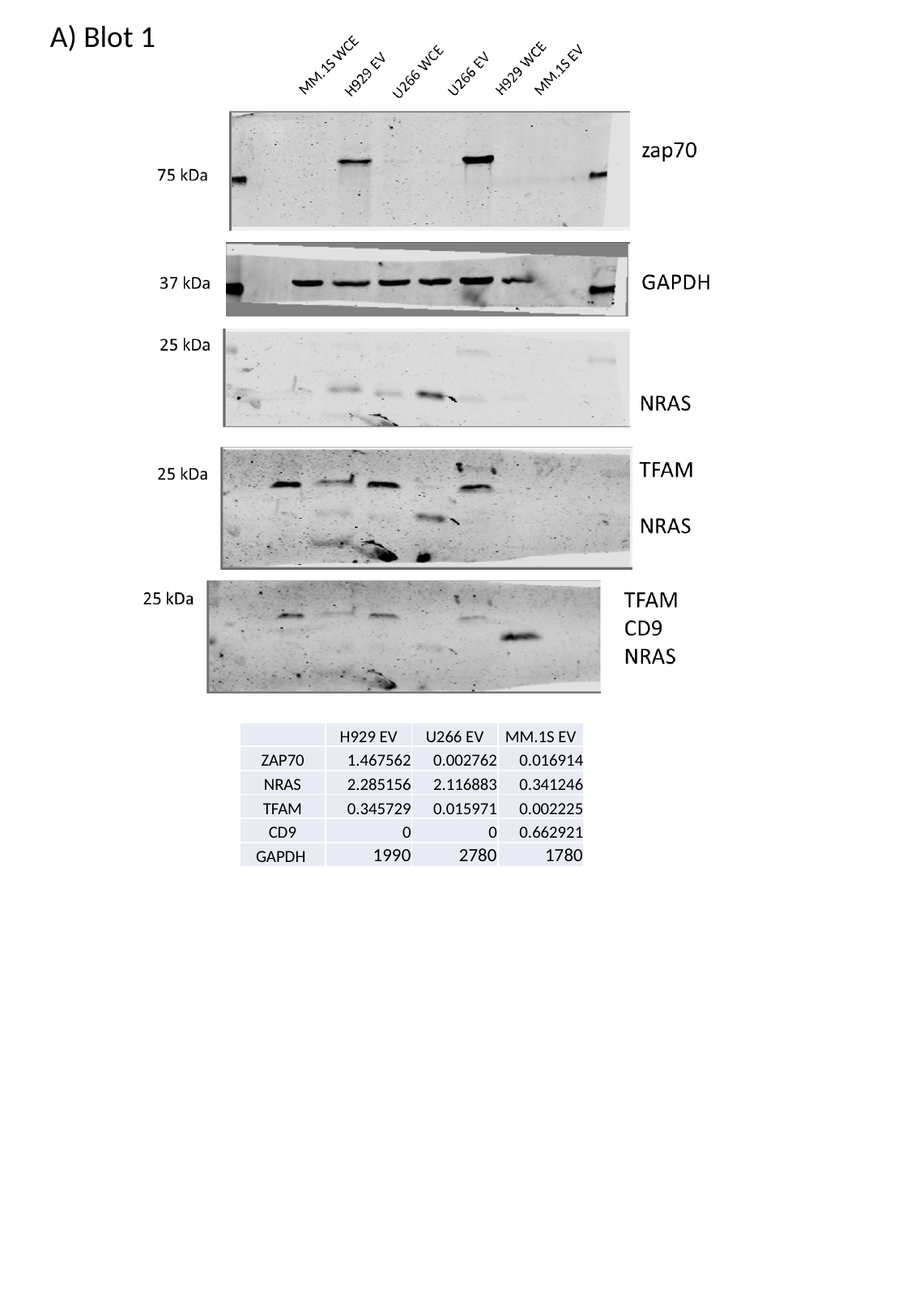

A) Blot 1
MM.1S EV
MM.1S WCE
| | H929 EV | U266 EV | MM.1S EV |
| --- | --- | --- | --- |
| ZAP70 | 1.467562 | 0.002762 | 0.016914 |
| NRAS | 2.285156 | 2.116883 | 0.341246 |
| TFAM | 0.345729 | 0.015971 | 0.002225 |
| CD9 | 0 | 0 | 0.662921 |
| GAPDH | 1990 | 2780 | 1780 |

## Slide 9
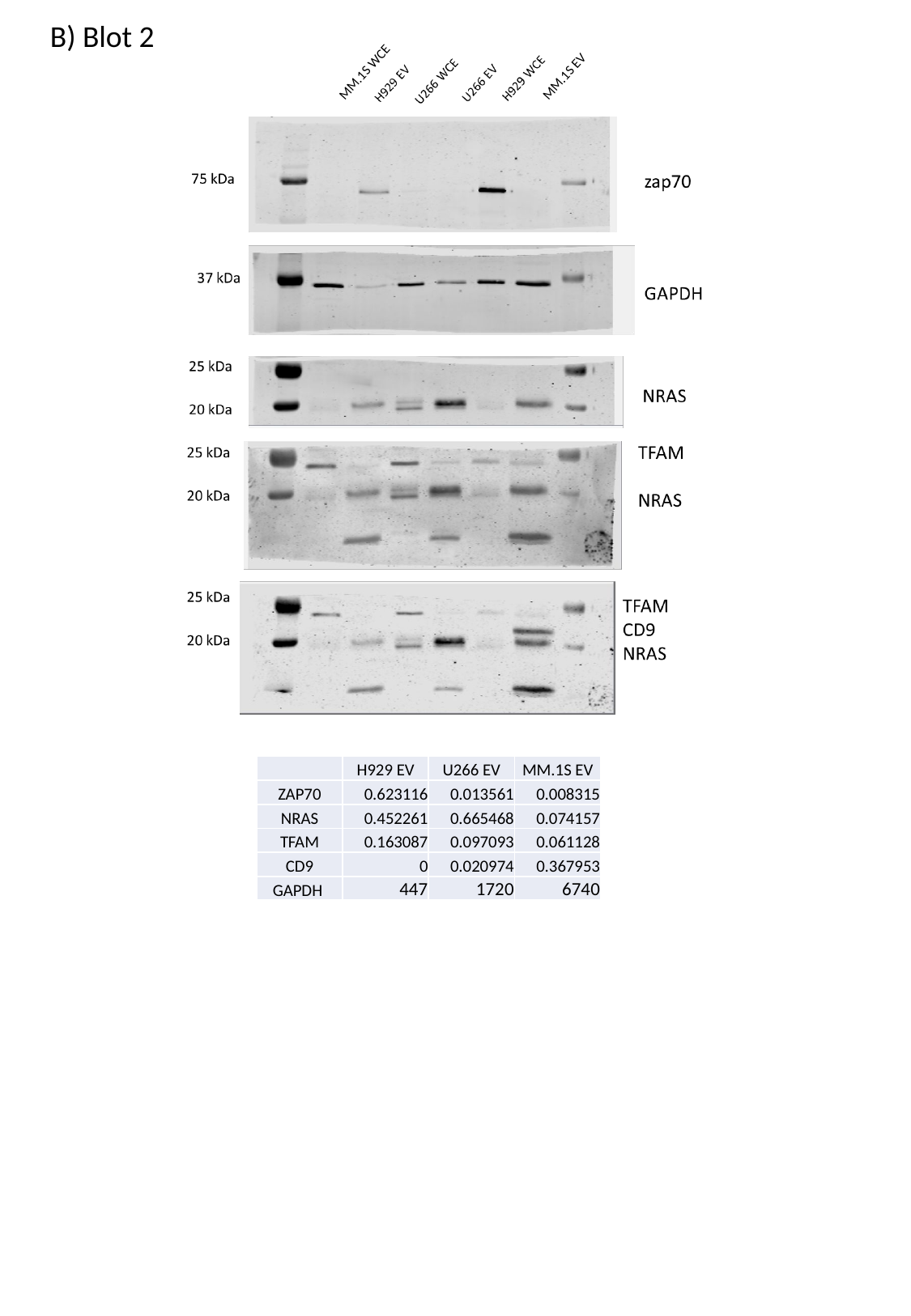

B) Blot 2
MM.1S EV
MM.1S WCE
| | H929 EV | U266 EV | MM.1S EV |
| --- | --- | --- | --- |
| ZAP70 | 0.623116 | 0.013561 | 0.008315 |
| NRAS | 0.452261 | 0.665468 | 0.074157 |
| TFAM | 0.163087 | 0.097093 | 0.061128 |
| CD9 | 0 | 0.020974 | 0.367953 |
| GAPDH | 447 | 1720 | 6740 |

## Slide 10
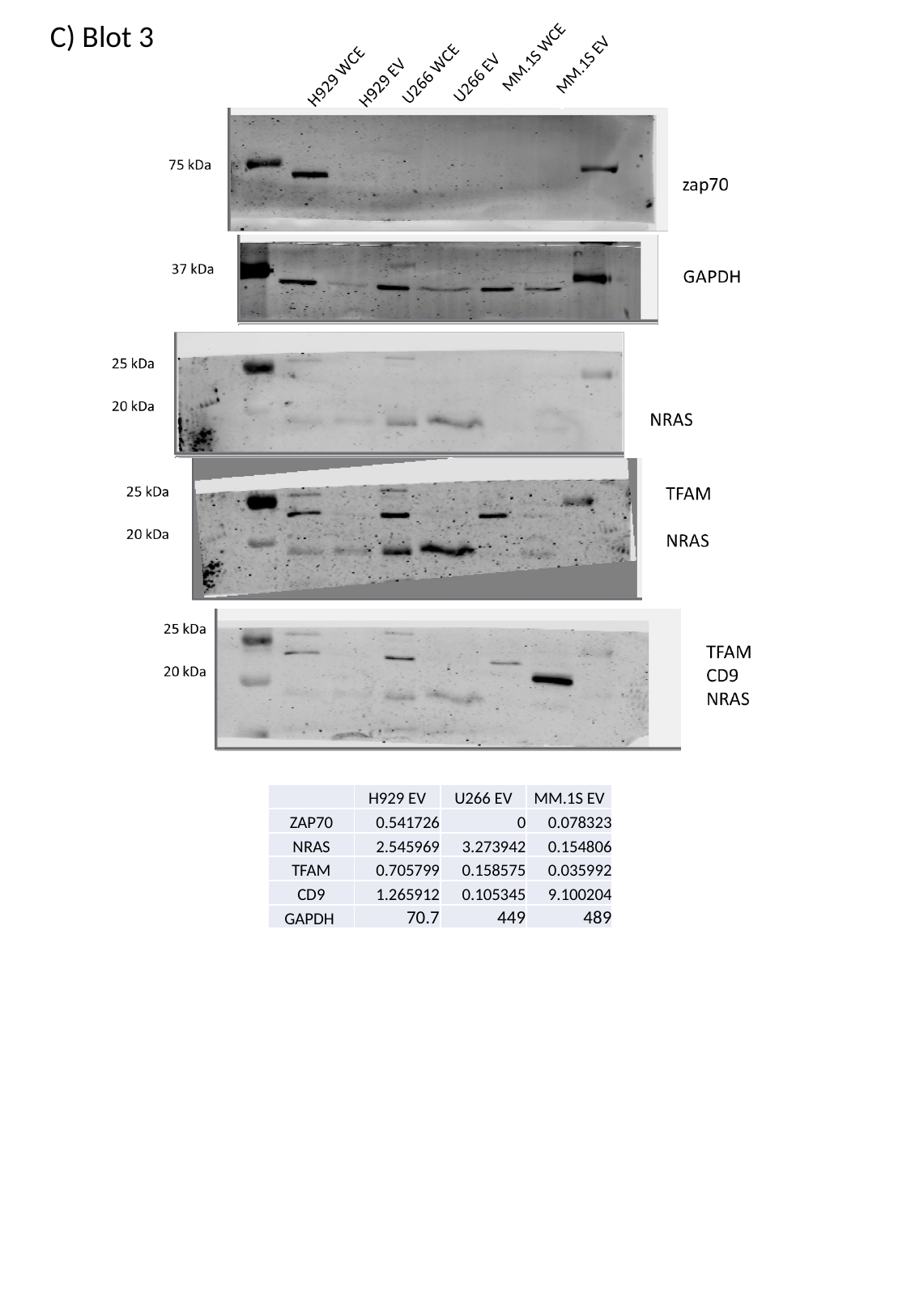

C) Blot 3
MM.1S WCE
MM.1S EV
| | H929 EV | U266 EV | MM.1S EV |
| --- | --- | --- | --- |
| ZAP70 | 0.541726 | 0 | 0.078323 |
| NRAS | 2.545969 | 3.273942 | 0.154806 |
| TFAM | 0.705799 | 0.158575 | 0.035992 |
| CD9 | 1.265912 | 0.105345 | 9.100204 |
| GAPDH | 70.7 | 449 | 489 |

## Slide 11
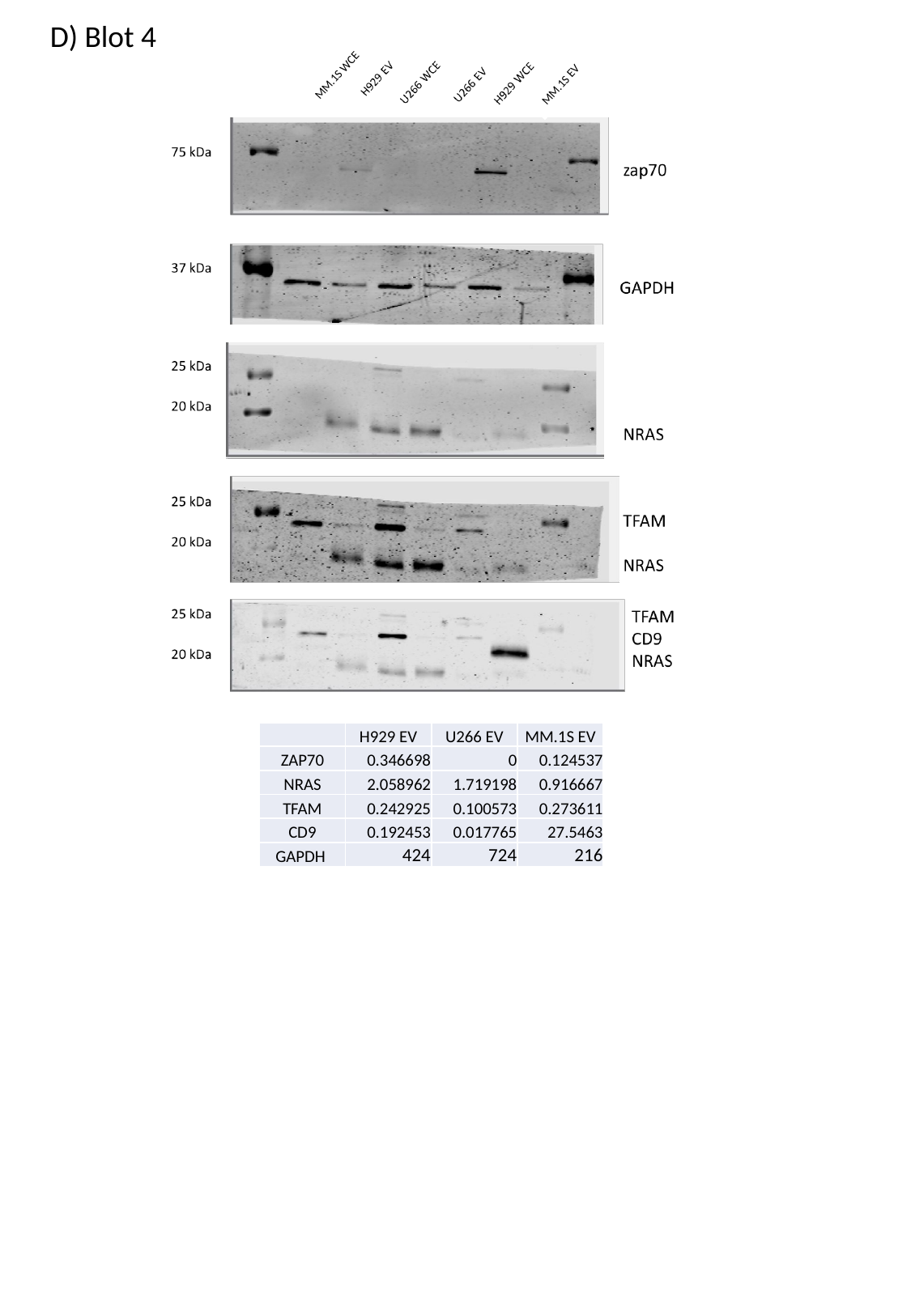

D) Blot 4
MM.1S WCE
MM.1S EV
| | H929 EV | U266 EV | MM.1S EV |
| --- | --- | --- | --- |
| ZAP70 | 0.346698 | 0 | 0.124537 |
| NRAS | 2.058962 | 1.719198 | 0.916667 |
| TFAM | 0.242925 | 0.100573 | 0.273611 |
| CD9 | 0.192453 | 0.017765 | 27.5463 |
| GAPDH | 424 | 724 | 216 |

## Slide 12
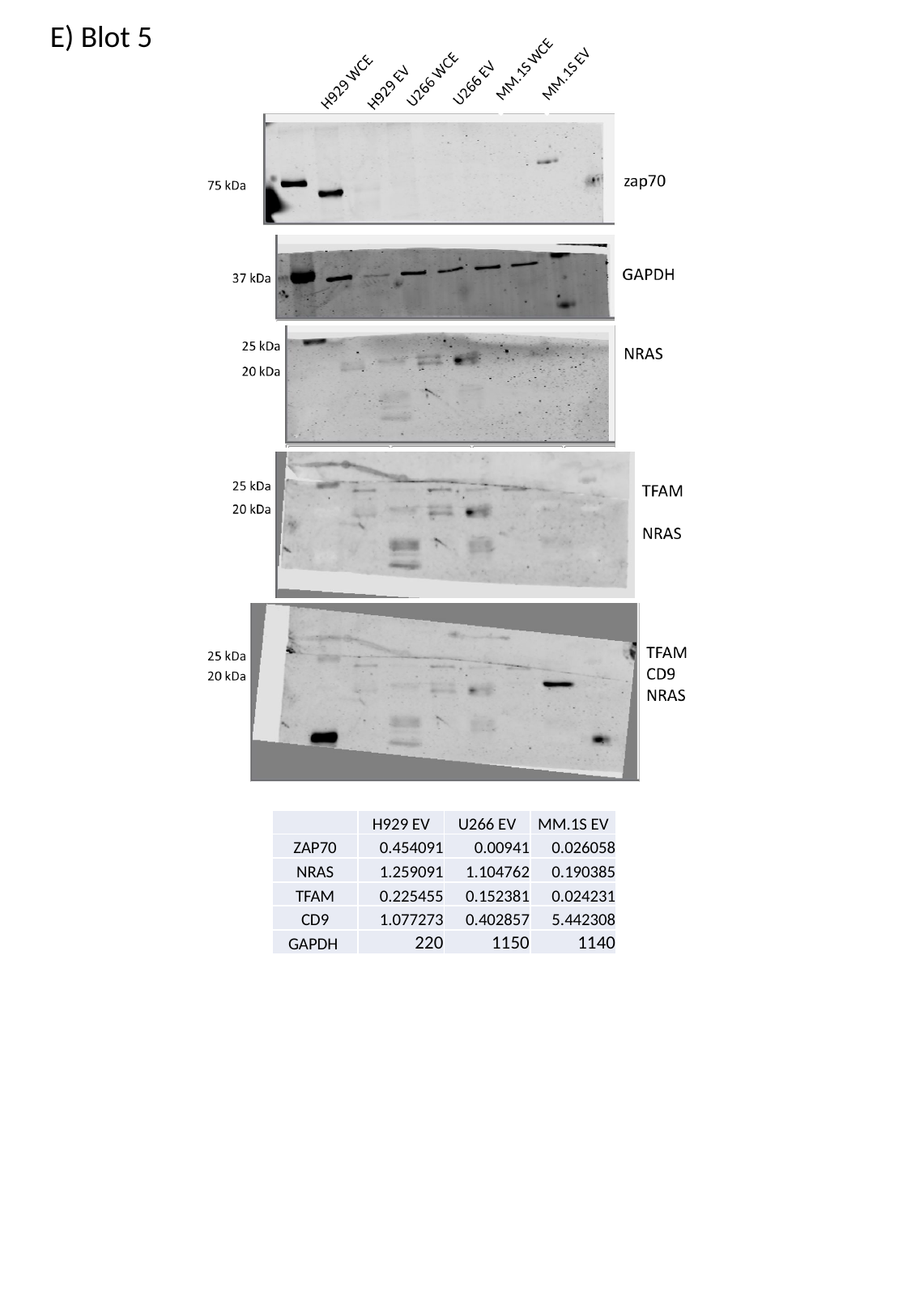

E) Blot 5
MM.1S EV
MM.1S WCE
| | H929 EV | U266 EV | MM.1S EV |
| --- | --- | --- | --- |
| ZAP70 | 0.454091 | 0.00941 | 0.026058 |
| NRAS | 1.259091 | 1.104762 | 0.190385 |
| TFAM | 0.225455 | 0.152381 | 0.024231 |
| CD9 | 1.077273 | 0.402857 | 5.442308 |
| GAPDH | 220 | 1150 | 1140 |

## Slide 13
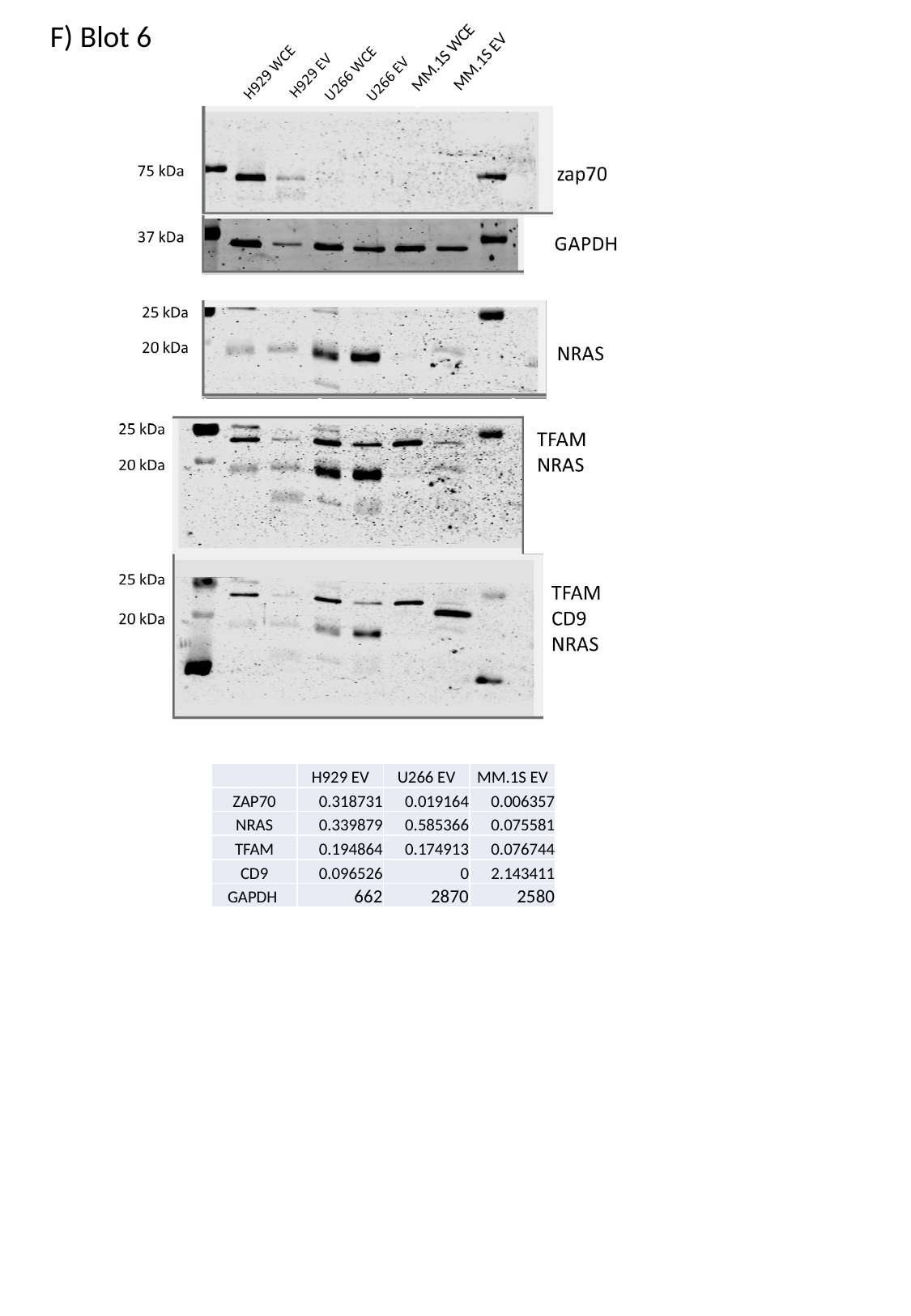

F) Blot 6
MM.1S EV
MM.1S WCE
| | H929 EV | U266 EV | MM.1S EV |
| --- | --- | --- | --- |
| ZAP70 | 0.318731 | 0.019164 | 0.006357 |
| NRAS | 0.339879 | 0.585366 | 0.075581 |
| TFAM | 0.194864 | 0.174913 | 0.076744 |
| CD9 | 0.096526 | 0 | 2.143411 |
| GAPDH | 662 | 2870 | 2580 |

## Slide 14
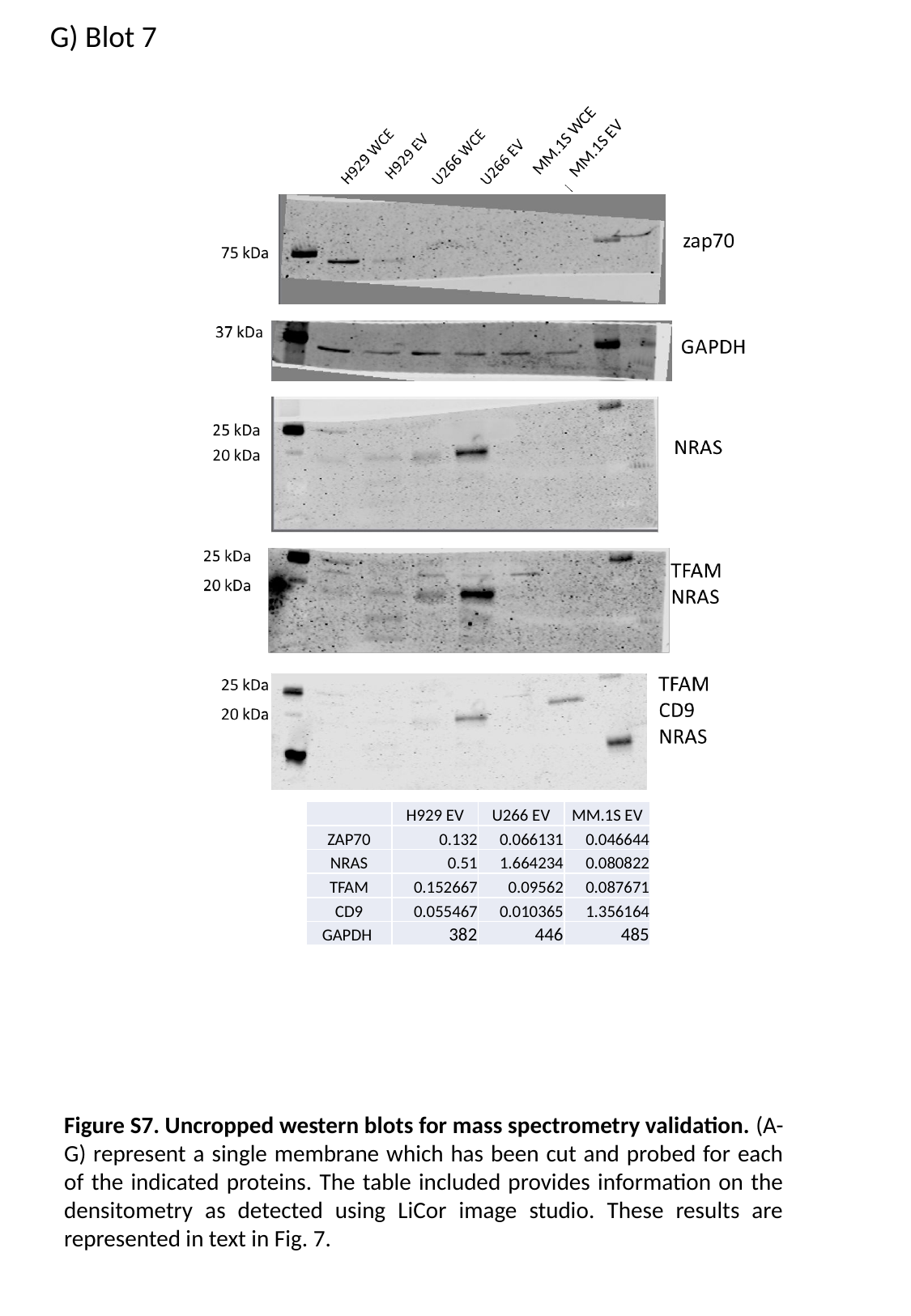

G) Blot 7
MM.1S WCE
MM.1S EV
| | H929 EV | U266 EV | MM.1S EV |
| --- | --- | --- | --- |
| ZAP70 | 0.132 | 0.066131 | 0.046644 |
| NRAS | 0.51 | 1.664234 | 0.080822 |
| TFAM | 0.152667 | 0.09562 | 0.087671 |
| CD9 | 0.055467 | 0.010365 | 1.356164 |
| GAPDH | 382 | 446 | 485 |
Figure S7. Uncropped western blots for mass spectrometry validation. (A-G) represent a single membrane which has been cut and probed for each of the indicated proteins. The table included provides information on the densitometry as detected using LiCor image studio. These results are represented in text in Fig. 7.
